# Correcting ligands, metabolites, and pathways

**DOI:** 10.1186/1471-2105-7-517

**Published:** 2006-11-28

**Authors:** Martin A Ott, Gert Vriend

**Affiliations:** 1 Centre for Molecular and Biomolecular Informatics, Nijmegen Centre for Molecular Life Sciences, Radboud University Nijmegen, P.O. Box 9010, 6500 GL Nijmegen, The Netherlands

## Abstract

**Background:**

A wide range of research areas in bioinformatics, molecular biology and medicinal chemistry require precise chemical structure information about molecules and reactions, *e.g. *drug design, ligand docking, metabolic network reconstruction, and systems biology. Most available databases, however, treat chemical structures more as illustrations than as a datafield in its own right. Lack of chemical accuracy impedes progress in the areas mentioned above. We present a database of metabolites called BioMeta that augments the existing pathway databases by explicitly assessing the validity, correctness, and completeness of chemical structure and reaction information.

**Description:**

The main bulk of the data in BioMeta were obtained from the KEGG Ligand database. We developed a tool for chemical structure validation which assesses the chemical validity and stereochemical completeness of a molecule description. The validation tool was used to examine the compounds in BioMeta, showing that a relatively small number of compounds had an incorrect constitution (connectivity only, not considering stereochemistry) and that a considerable number (about one third) had incomplete or even incorrect stereochemistry. We made a large effort to correct the errors and to complete the structural descriptions. A total of 1468 structures were corrected and/or completed. We also established the reaction balance of the reactions in BioMeta and corrected 55% of the unbalanced (stoichiometrically incorrect) reactions in an automatic procedure. The BioMeta database was implemented in PostgreSQL and provided with a web-based interface.

**Conclusion:**

We demonstrate that the validation of metabolite structures and reactions is a feasible and worthwhile undertaking, and that the validation results can be used to trigger corrections and improvements to BioMeta, our metabolite database. BioMeta provides some tools for rational drug design, reaction searches, and visualization. It is freely available at  provided that the copyright notice of all original data is cited. The database will be useful for querying and browsing biochemical pathways, and to obtain reference information for identifying compounds. However, these applications require that the underlying data be correct, and that is the focus of BioMeta.

## Background

The importance of knowledge about metabolites for understanding life is well demonstrated by their prominent role in the Kyoto Encyclopedia of Genes and Genomes [1-5], MetaCyc[6], the Boehringer-Mannheim charts[7,8], Brenda[9,10], ExPASy[11], ChEBI[12], or PubChem[13]. These databases vary considerably in their focus. Some have a strong emphasis on enzymatic information, while others are metabolic databases containing, for example, information about metabolites, reactions, enzymes, and genes. Most of these systems also contain a limited number of small xenobiotic compounds.

Three frequently used pathway databases are KEGG, MetaCyc, and Brenda. KEGG is a suite of databases and associated software, interlinking data on small compounds, reactions, enzymes, and genes. The graphical pathway maps to which the databases are linked are an important feature of KEGG. MetaCyc[6] is a curated database of experimentally elucidated metabolic pathways from many organisms. It contains data about pathways and their associated small compounds, enzymes, and genes. KEGG and MetaCyc both contain data on metabolites; unfortunately, MetaCyc does not hold atomic information on small compounds. The metabolite data in KEGG (the Compound section of the Ligand database) have been organized such that they are easily downloadable as chemical structure files in the MDL molfile format[14].

The Boehringer-Mannheim wall charts[7] offer a glimpse on the enormous complexity of the interlinked metabolic network. The small-molecule part of these charts has been extracted into a C@rol[15] database called BioPath[16]. Brenda[10] is a curated enzyme database that provides pictures of reaction diagrams and chemical structures of small compounds. ChEBI[12] is a dictionary of molecular entities focusing on small compounds. PubChem[13] is a database of chemical structures of small compounds and information on their biological activities. Many of these databases, especially ChEBI and PubChem, contain cross-references to other databases, notably KEGG. PubChem merely lists these references, but in ChEBI the entries are curated and classified using a chemical ontology.

Even though the systems mentioned above provide a wealth of data, they cover only a very small portion of all possible metabolites. Estimates on the total number of metabolites range from 200,000[17] to about 1,000,000[18], but even this higher estimate may be conservative. If plant and bacterial secondary metabolites (metabolites that are not necessary to keep the organism alive) are included then the numbers are enormously larger. The probable number of metabolites is also considerably larger than the number of corresponding genes[19], so it seems that the currently available databases cover at best 2% of the total number of metabolites. Of course, this discussion includes only metabolites from biochemical pathways, not the catabolism of xenobiotics – the number of small compounds involved in those processes may go up indefinitely as many thousands of xenobiotics are being developed every year.

The limited availability of metabolite data stands in marked contrast to the high demand for them. A wide range of research areas in bioinformatics, molecular biology, and medicinal chemistry require chemical structure information about molecules and reactions. This need is best seen for fields like total synthesis of natural products, drug design, ligand docking, metabolomics, metabolic network reconstruction, or systems biology. Metabolites have been used in several ways in drug design. First, endogenous human metabolites can be used as leads in drug design. Second, many metabolites from plants or other sources are medicines or good leads for drug design[20]. All such applications require the molecular information to be correct, complete, and accurate. We have therefore set out to design and implement BioMeta, a database that aims at providing correct metabolite structures and correct reactions. The philosophy behind the correction principles is that enzymes cannot invent new chemistry; they can only speed up existing chemistry. So, if a metabolic conversion does not make sense from an organic chemistry point of view, it also does not make sense from a metabolic point of view.

Structure descriptions of compounds can be checked automatically for incorrect valences and undefined stereocenters, and reactions can be checked automatically for incorrect stoichiometry. Once a structure description is administratively correct and completely defined, further error checking (incorrect composition, connectivity, or stereochemistry) will require manual inspection and comparison to other sources, *e.g., *original references and other compounds related to it through known reactions. However, even for the automatic validations, no general tools are currently available, so we developed them specially for BioMeta.

BioMeta is a relational database containing information about known metabolites and the validation of their structures. It also holds metabolic reactions. It is based entirely on freely available metabolite data (mainly from KEGG) and is freely available as a web service[21] (provided that the copyright notices of the original data providers are respected).

## Construction and Content

### BioMeta database design

**Figure 1:**
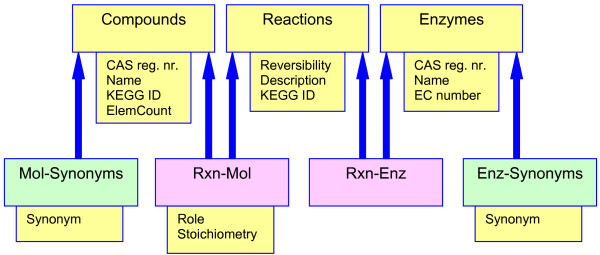
**Outline of the BioMeta database design, showing the main tables and the most important link tables**. The main tables are Compounds, Reactions, and Enzymes. The most important link tables are displayed in purple. The "green" tables contain synonyms (for both compounds and enzymes). For clarity, data fields of lesser importance have been omitted.

The main ideas behind BioMeta's database design are similar to those in the KEGG Ligand database. BioMeta's major tables hold compounds (molecules), reactions, enzymes, and references (literature and other sources). A series of relation tables connect these elementary data. Two relations are pivotal: 1) reactions are described in terms of participating molecules (and a molecule has a particular role in a reaction); and 2) enzymes catalyze one or more reactions (and a reaction is catalyzed by one or more enzymes). No direct relation exists between compounds and enzymes – they are only linked indirectly through reactions. At present, only two roles are used: reactants and products – these are simply the compounds on the left- and right-hand sides of the reaction arrow. (Note that the term "reactant" appears to be used differently by chemists and biologists. Chemists use it as a synonym for the rarely used term "educt"; some biologists seem to use it to indicate "either substrate or product". We avoid the term "substrate" since both reactants and products can be substrates of an enzyme, and the term loses its meaning if the reaction is not catalyzed.) The database design allows compound roles such as inhibitor and activator to be added easily. Figure 1 shows an outline of the database design and the most important data tables. Compounds and enzymes have much in common, so both tables contain similar data fields: CAS registry number, (common) name, systematic name, references to other databases (be it KEGG accession numbers or EC numbers). PostgreSQL does not allow arrays of values (multiple values) for a given data field. For each such field a separate table must exist which is linked (through the entry IDs) to the corresponding main table. Since both compounds and enzymes usually have a number of different names, these synonyms are stored in separate synonym tables. For both compounds and enzymes, there is a second synonym table (not shown in Figure 1) containing so-called "fuzzy" synonyms in which are non-alphanumeric characters have been removed and all letters have been converted to upper case. These extra tables allow "fuzzy" synonym searches.

The reactions table contains information pertaining to reactions as a whole, such as reversibility, balance, or the KEGG accession number. The relations between molecules and reactions are stored in the Rxn-Mol link table, each row in this table describing the role (reactant, product) and stoichiometry of a particular molecule in a particular reaction. The relations between reactions and enzymes are stored in the Rxn-Enz link table; each row in this table indicates that a particular enzyme catalyzes a particular reaction. The database does not contain other information about pathways or pathway maps, nor does it contain gene, species, or cellular localization information.

An additional data table (not shown in Figure 1) is used to store molecular formula information. This table contains the appropriate coefficient for each compound/element combination (*e.g.*, the 2 in H_2_O). The field ElemCount in the Compounds data table contains the number of different elements in the formula of a compound. In combination, they allow formula searches such as "all compounds with twenty carbon atoms and at least 38 hydrogen atoms and at most three different elements".

### Compounds and reactions in the KEGG Ligand database

The KEGG metabolic pathways are graphical maps displaying compounds and reactions from the Ligand database [1-4]. This Ligand database is tightly coupled to the KEGG pathway maps. It consists of three sections: Compound, Reaction, and Enzyme. The Compound section contains about 13,000 small compounds, most of which are involved in enzymatic reactions as substrates, products, cofactors, or inhibitors. A number of drugs and xenobiotics have also been included but these are currently being transferred to a separate Drug section in the KEGG Ligand database. Each compound entry contains an ID code, CAS registry number, common name, synonyms, systematic name, chemical formula, structure as an MDL molfile[14] with a GIF image, reaction links, and enzyme links. The Reaction section contains about 6,500 reactions. Each reaction entry contains an ID code, name of the enzyme, a textual description of the reaction, chemical structures of the substrates and products as an MDL rxnfile[14] and as a GIF image, an equation expressed in compound ID codes, links to Enzyme entries, and a link to the corresponding KEGG pathway map. The rxnfiles are constructed from the molfiles of the participating compounds. The Enzyme section (about 4,500 entries) contains the enzymes, indexed by their EC number. The majority of entries (compounds, reactions, and enzymes) in BioMeta were obtained from KEGG.

**Figure 2:**
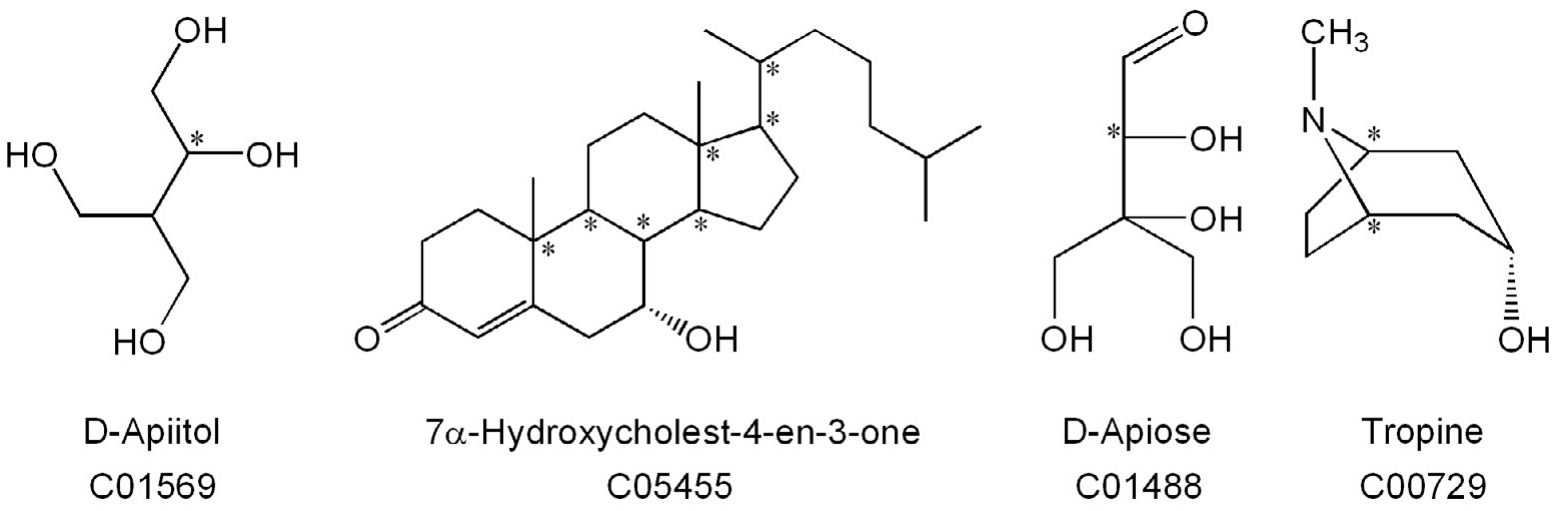
**Sample structures from KEGG with incomplete stereochemistry**. Undefined stereocenters are indicated by an asterisk. C01569: undefined; C05455: defined by convention (cholestane skeleton); C01488: defined using Fischer projection; C00729: defined using perspective drawing.

We obtained the compounds from the KEGG Ligand database as molfiles. These molfiles contain structural information in a so-called 2D representation, meaning that the drawings are primarily intended to show the constitution (connectivity) of the molecules; 3D information is absent. Hydrogen atoms are usually omitted unless they are used to indicate the stereochemical configuration. The configuration of stereocenters is indicated using wedged and dashed bonds as is common in organic chemistry. In principle, these 2D structure representations are sufficient for the chemical identification of compounds. Unfortunately, not all structures are provided with stereochemical detail. Four examples of commonly observed deviations are shown in Figure 2. Sometimes the configuration of a stereocenter is omitted (*e.g.*, C01569). The stereochemistry of the base skeleton is sometimes left out because it is considered to be commonly known (*e.g., *steroids such as C05455). In a number of structures (mostly carbohydrates such as C01488) the stereochemistry is described using a Fischer projection. In other cases a perspective drawing has been used (*e.g.*, C00729). While these different styles of representation can usually be correctly interpreted by a knowledgeable chemist, they have no meaning within the molfile format, and any software processing such molfiles cannot function reliably. In particular, a 3D model building program would assign random configurations to undefined stereocenters; or worse, that software might crash.

Lack of stereochemical completeness may also prevent database normalization. When a compound is entered in a relational database, duplicate checking must prevent redundant entries. If the new structure is actually the same as one already present in the database but it is not completely described, the duplicate check is likely to fail and a new compound entry is wrongly introduced. In the case of metabolic modeling, incomplete or erroneous networks may be built because the chemical identity of two compounds from different reactions goes undetected.

**Figure 3:**
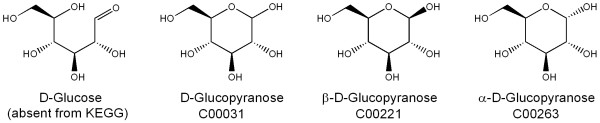
**Different variants of D-glucose with their names and KEGG accession codes**. The α – and β-D-glucopyranose forms account for 37% and 63% respectively of the equilibrium mixture in aqueous solution. The open form, absent from KEGG, is present in small amounts. The D-glucofuranose forms (five-membered rings) are not shown, as they are only present in insignificant amounts. The fact that C00031 represents the equilibrium mixture of C00221 and C00263 causes a problem in consistency of database management of these compounds.

Even when chemical structures are represented correctly and completely, structure representation may be complicated because in physical reality a compound may consist of a dynamic mixture of rapidly interconverting structures. Two important types of such behavior are tautomerism and anomerism. In the case of tautomerism, acidic hydrogen atoms may wander freely over basic sites. The imidazole ring in histidine is a familiar example. Anomerism, which is common with carbohydrates, is the reversible opening and closing of ring forms (mainly pyranoses and furanoses). The ring forms, which predominate in solution, may exist in two different stereoisomeric forms called alpha and beta (Figure 3). The treatment of tautomerism and anomerism is far from trivial and will be discussed in a separate publication.

We obtained the reactions from the KEGG Ligand database in the form of an ASCII file. This file does contains neither information about reversibility nor, if irreversible, about the direction of the reactions. Reversibility/direction information is obtained from a separate ASCII file which KEGG maintains in connection to their graphical maps. Another important issue is the reaction balance that indicates whether an equal number of atoms of the various elements and an equal number of charges is present on both sides of the reaction arrow. The KEGG Reaction section of the Ligand database contained 6089 reactions, of which 5323 were provided with fully described and non-polymeric structures. The other 766 reactions either had missing structures (*e.g.*, "acceptor" or "phosphorylated protein") or involved polymeric compounds (*e.g.*, "oligopeptide" or "starch"), preventing assessment of their balance. We found that 3711 reactions were balanced and that 1612 were unbalanced. Unbalanced reactions can obviously not be used for the automatic construction of reaction networks as is done in metabolic modeling and systems biology. It is an easy matter to identify the unbalanced reactions, but a major problem to correct them. The cases where just a simple component such as H^+^, H_2_O, CO_2_, or H_3_PO_4 _is missing could be amenable to automatic correction. Most cases, however, will require tedious manual correction. Using an automatic procedure, we have corrected the reactions where the "imbalance" was H_2_O, H^+^, or 2H^+^, accounting for 893 reactions (55% out of 1612) reactions. Limited resources have prevented us from making a more thorough attempt.

### Chemical structure validation software

Many biologists, bioinformaticians, and other researchers in related areas usually identify a compound by name. To chemists, the identity of a compound is normally determined by its 2D structure. Incorrect 2D structures cannot be linked to actual chemical species, and incomplete ones (those lacking full stereochemical detail) cannot be linked to a unique one. We have written validation software that checks the correctness and completeness of structure descriptions (*i.e.*, molfiles) of small compounds. It performs the following tasks:

1. Determining and checking valency;

2. Ring and aromaticity detection;

3. Calculation of molecular formula, weight, and exact mass;

4. Stereochemistry detection;

5. Canonicalization;

6. Calculation of canonical string identifiers.

MDL molfiles describe 2D chemical structures in a valence-bond representation. Valences can therefore be checked using the Lewis structure concept (*i.e.*, the number of electrons in the valence shell of first-row elements is usually eight and can only be less, never more). As a rule, the structures are hydrogen-suppressed (hydrogen atoms occur only when needed to indicate stereochemical configurations), so the valence detection will give the numbers of (implicit) hydrogen atoms on each atom which, of course, needed for the calculation of the molecular formula and weight.

**Figure 4:**
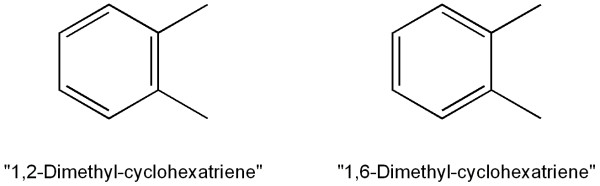
The two Kekulé structures (mesomeric forms) for ortho-xylene.

Rings are detected primarily to be able to detect aromaticity. Without aromaticity detection, the two Kekulé structures for ortho-xylene would be considered isomeric (Figure 4). Aromaticity detection was restricted to benzene-type rings (pyridine, pyrimidine, etc.) and pyrrole-type rings (thiophene, imidazole, oxazole, etc.) and all their fused combinations.

**Figure 5:**
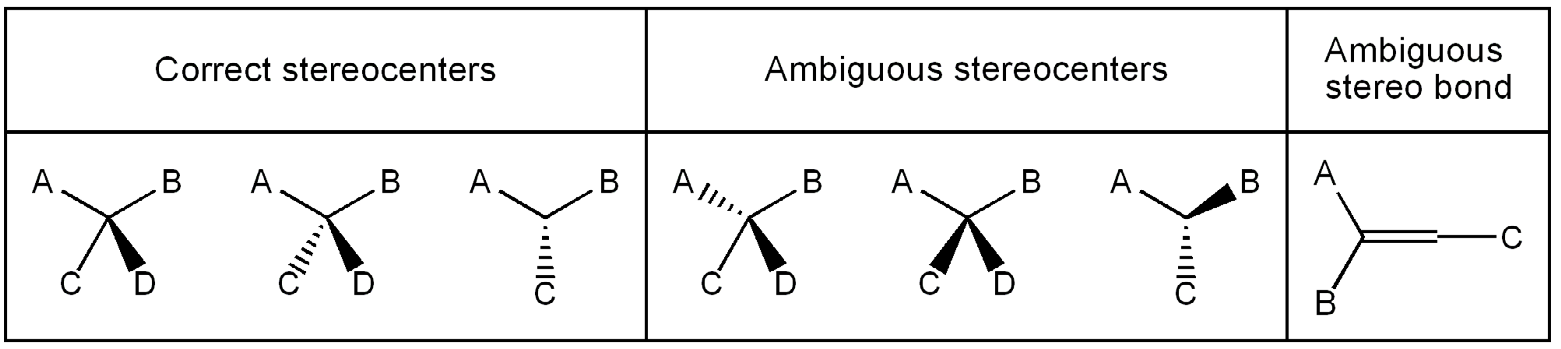
Valid and invalid (ambiguous) representations of sp3 (tetrahedral) stereochemistry and double bond stereochemistry.

All carbon, nitrogen, and phosphorus atoms having four single bonds (or three plus one to an implicit hydrogen) are treated as potential stereocenters. An atom is a stereocenter if its inversion would change the molecule into a different stereoisomer (determined by the canonicalization routine described below). If it is not a stereocenter, any stereo bonds (wedges or dashes) on it are ignored; if it is, its configuration is determined based on the stereo bonds present (the absence of such bonds indicating an undefined stereocenter). Note that not all arrangements of stereo bonds around a center are meaningful (Figure 5).

Similarly, C = C, C = N, and N = N double bonds were examined for possible cis/trans isomerism, excluding aromatic double bonds and those in cumulenes such as allenes. A bond is a stereo double bond if its "inversion" (cis-trans isomerization) would change the structure into a different stereoisomer. The 2D coordinates suffice for establishing the configuration. Only if one of the atoms on the bond is singly substituted and the bond angle at that atom is 180 degrees can the stereochemistry of a double bond remain unknown, *i.e.*, undefined (Figure 5). Finally, the program determines whether the molecule is chiral. A molecule is chiral only if it is not superimposable onto its mirror image. The mirror image is easily obtained by inverting all stereocenters. If the mirror image is not identical to the original molecule (determined by the canonicalization routine described below), then the molecule must be chiral. If the structure in a molfile is chiral, the intended structure may be the enantiomer as it has been drawn (absolute stereochemistry) or it may be the racemic mixture of that structure (relative stereochemistry) or, perhaps, a single but unknown enantiomer. In the molfile this is indicated through the so-called "chiral flag"[14] which is set to 1 in the case of absolute stereochemistry. If a structure is chiral, but the flag has not been set to 1 in the molfile, the validation program issues a warning – since for the purpose of a biochemical database, the intended structure is expected to be a single, known enantiomer.

**Figure 6:**
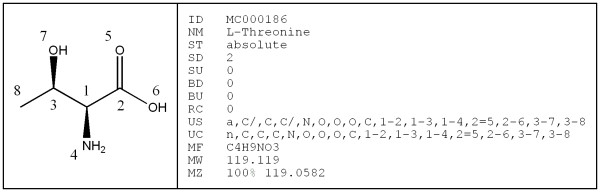
**Canonically numbered structure and calculated data fields for L-threonine**. NM = (common) name, ST = absolute/relative stereochemistry, SD = number of defined stereocenters, SU = number of undefined stereocenters, BD = number of defined double bonds, BU = number of undefined double bonds, RC = number of rings, US = unique string (stereochemistry included), UC = unique string (stereochemistry excluded), MF = molecular formula, MW = molecular weight, MZ = M/Z peak with abundance.

Canonicalization is the unique numbering of atoms in a molecular structure. It helps to uniquely identify a molecule, independently of how it is drawn. We implemented a canonicalization method based on the Morgan algorithm[22] similar to the SEMA (stereochemically extended Morgan) algorithm[23]. Canonicalization and stereochemistry detection are performed simultaneously because the identity of two molecular representations may have to be assessed during stereochemistry detection (see the preceding section). The canonicalization routine generates a string that can be used for text-based identity checking and hence for structure matching. This "unique" string is similar in nature to strings such as the SEMA name[23], unique Smiles[24], PRODRG molecular descriptor string[25], and InChI[26]. A second "unique" string is calculated the same way but neglecting stereochemistry. This second string can be used to search for stereoisomers. Figure 6 shows the canonically numbered structure of L-threonine and a number of calculated data fields such as the number of stereocenters and double bonds, the unique strings mentioned above, the molecular formula and weight, and the M/Z peak based on 100% abundance of the most common isotopes.

### Validation of compounds and reactions from the KEGG Ligand database

BioMeta was intended to be complementary to the KEGG Ligand database by focusing on the application of organic chemical knowledge to small compounds, thus ensuring that the compounds and implicitly the reactions are correct. Hundreds of molecular structures were corrected or improved. Table 1 gives a breakdown of the validation results and the corrections made in the 12,815 molecule entries present in both BioMeta and the KEGG Ligand compound section of October 25, 2005. The validation program can detect only syntactical problems, *e.g.*, valence violations, undefined enantiomer, or invalid stereochemistry. Some are real errors requiring correction, such as a missing structure (if it is not polymeric or generic), valence violations, or ambiguously drawn stereocenters. Problems in the "undefined" categories suggest incomplete structural information, but not all such cases are necessarily incorrect, *e.g.*, a drug that is a racemic compound would trigger the warning "unspecified enantiomer". Problems in the "incorrect" categories have not been detected by the validation program since these errors are semantic rather than syntactic – they were detected through visual inspection. A total of 1468 structures were corrected. The large majority of valence errors involved nitrogen atoms that were not trivalent. The most common of these were: 1) a nitrogen atom having one double bond and two single bonds, but no charge (*i.e.*, intended to be a pyridinium- or nitro-type nitrogen), these were corrected by removing an attached hydrogen or else by adding a positive charge, and 2) coordinative bonds from a imine-type nitrogen to a metal indicated as covalent. Unfortunately, the molfile format[14] does not support coordinative bonds, so these bonds had to be removed. Table 2 gives a more detailed breakdown of the sp^3 ^stereochemistry enhancements from Table 1 (the numbers are slightly different because double-bond stereochemistry is omitted). In Table 2 the "unspecified enantiomer" cases from Table 1 are split between two "relative" stereochemistry cases, incompletely and completely defined. All cases (also for meso compounds) are listed so that the numbers add up.

**Table 1:** Detected and corrected problems in the BioMeta database

**Type of Problem**	**# in KEGG**	**# in BioMeta**	**# Corrected**
Structure missing	1239	1106	133
Valence violation(s)	76	0	76
Incorrect constitution	unknown	unknown	107
**Total (constitution)**	**1315**	**1106**	**316**
			
Undefined stereo double bond(s)	35	32	3
Invalid sp3 stereocenter(s)	70	47	23
Ambiguous sp3 stereocenter(s)	46	0	46
Undefined sp3 stereocenter(s)	1398	865	533
Unspecified enantiomer	2326	1840	486
Undefined sp3 stereochemistry	554	366	188
Incorrect stereochemistry	unknown	unknown	69
**Total (stereochemistry)**	**3990**	**2907**	**1152**
			
**Total corrected**			**1468**

The table shows the validation and correction results of 12,815 entries present in both the KEGG Compound (version of October 25, 2005) and BioMeta databases. Note that the absence of a structure does not need to be an error – it may be a generic compound such as "acceptor" or "phosphorylated protein". Likewise, not all "unspecified enantiomer" cases need to be errors – a number of drugs may be racemic compounds. The row "total (stereochemistry)" is not the sum of the preceding cases because compounds may have multiple problems. The rows with the totals do not add up because of the "unknown" entries – if these numbers were known then the numbers *would *add up.

**Table 2:** Statistics of sp^3 ^stereochemical content in the KEGG Compound and BioMeta databases

**Stereochemistry**	**OK**	**# in KEGG**	**# in BioMeta**	**# Corrected**
Not possible	+	3725	3764	
Undefined (*i.e.*, omitted)	–	554	366	188
Incompletely defined – meso	–	24	3	21
Incompletely defined – absolute	–	1080	691	389
Incompletely defined – relative	–	294	171	123
Completely defined – meso	+	56	89	
Completely defined – absolute	+	3735	4823	
Completely defined – relative	–	2032	1669	363
**Total not OK**		**3984**	**2900**	**1084**
**Total OK**		**7516**	**8676**	
**Total**		**11500**	**11576**	

The numbers in this table give a more detailed breakdown of the sp^3 ^stereochemistry enhancements from Table 1. Here "OK" means a single, completely defined, compound. The "unspecified enantiomer" cases from Table 1 are split here between two "relative" stereochemistry cases, incompletely and completely defined. Note that not all "Completely defined – relative" cases need to be errors – a number of drugs may be racemic compounds.

We also assessed the balance (stoichiometry) of the reactions. BioMeta contains 5323 reactions with fully described and non-polymeric structures, of which 3711 were balanced and 1612 were unbalanced. We also determined the "imbalance" of these reactions and those for which the imbalance was H_2_O, H^+^, or 2H^+ ^were corrected, accounting for 893 reactions (55% out of 1612) reactions. Limited resources prevent us from making a more thorough attempt.

KEGG version 3.6 contained the reaction "Fe + O_2 _+ 4 H^+ ^<=> Fe + 2 H_2_O" which prompted us to manually review all metal cations in the database. A number of those were present as "generic" cations, without an actual charge specification. To remedy this situation, six metal cations having definite oxidation states (Mn^3+^, Mn^2+^, Fe^3+^, Fe^2+^, Co^3+^, and Cu^+^) were added. Co^2+ ^and Cu^2+ ^were already present in KEGG. In the meantime, KEGG has also carried out this correction for the iron cations (in version 3.8) but not for manganese.

A variety of methods was used to determine the correct or intended structure. The name often provided sufficient information, but in many cases the reactions in which a compound was involved had to be consulted; either in the KEGG database or in other databases such as Brenda[9,10], MetaCyc[6], or ExPASy[11]. In the cases where database information was insufficient and the original literature had to be consulted. Brenda proved most useful for obtaining those references. We will discuss three examples of database corrections to illustrate the kinds of problems encountered, but also to illustrate the importance of these corrections for, *e.g.*, systems biology.

### Examples of validations and corrections

#### Example 1

**Figure 7:**
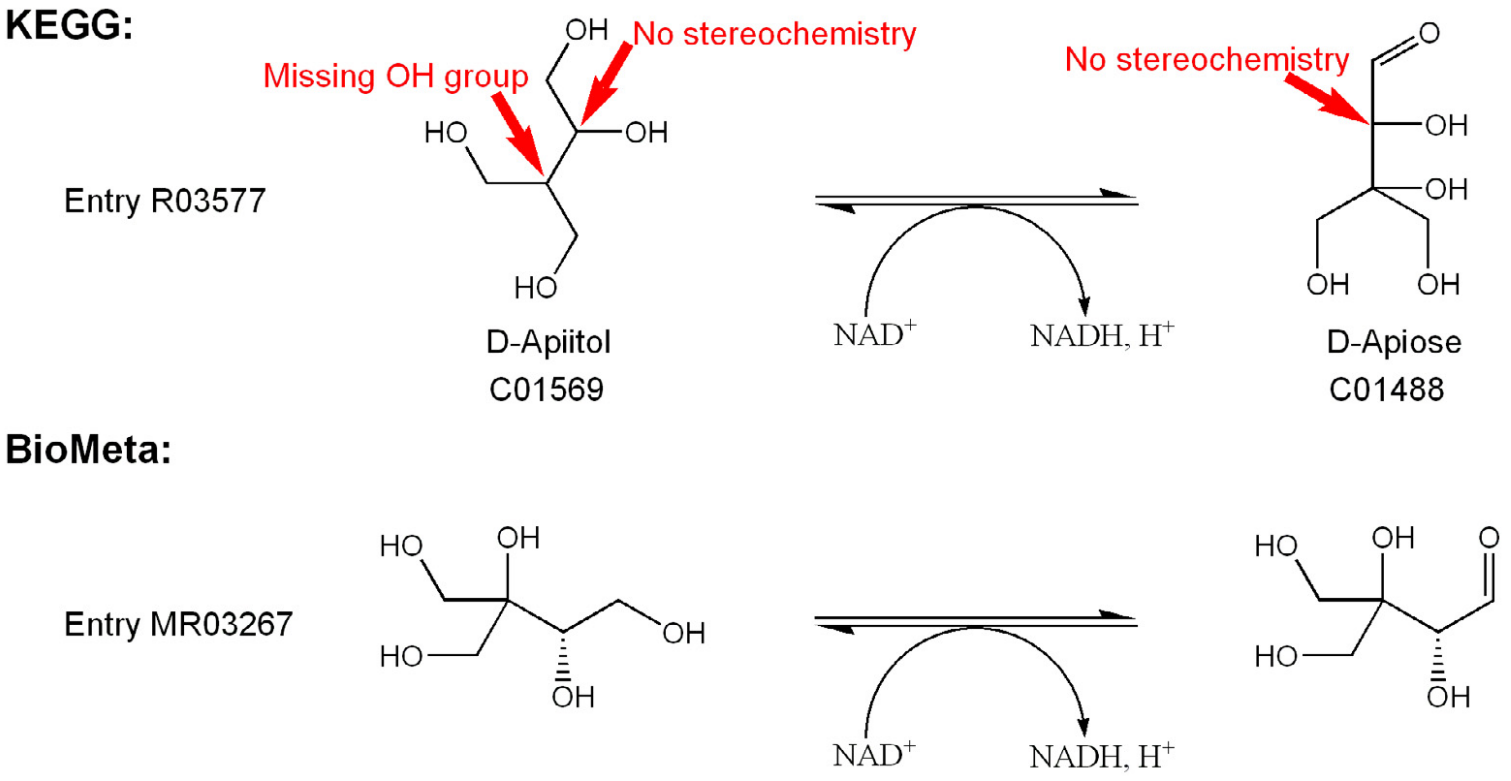
**KEGG reaction entry R03577 with corrected structures**. The configurations of the stereocenters were inferred from the names of the compounds. Note that the branched carbon in D-apiitol (C001569) is symmetrically substituted and therefore not a stereocenter. This remains the case after addition of the hydroxyl group.

Reaction entry R03577 from KEGG (Figure 7) is the reversible reduction of D-apiose (C01488) by NADH to give D-apiitol (C01569, see also Figure 2). The reaction itself is correct, but the structures are stereochemically undefined. Moreover, the structure of C01569 is wrong – it lacks a hydroxyl group at the branched carbon, which is only apparent after inspection of the reaction and comparison to D-apiose. Alternatively, a name search for apiitol in either the Beilstein[27] or CAS [28] databases will confirm the correct structure. To establish the intended stereochemistry, the prefixes "D-" in the compound names suffice.

#### Example 2

**Figure 8:**
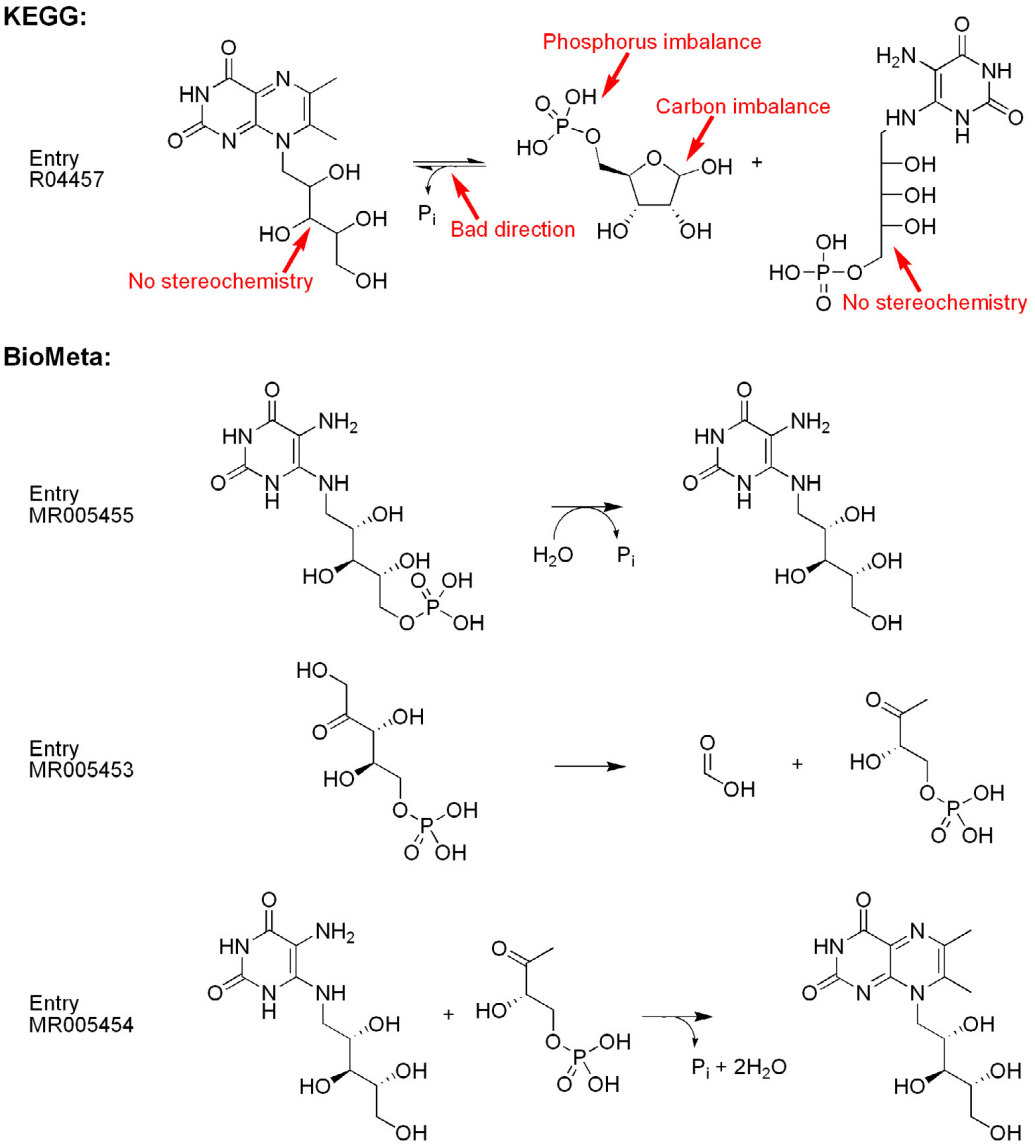
**The biosynthesis of 6,7-dimethyl-8-(1-D-ribityl)-lumazine from 5-amino-6-(5-phosphoribitylamino)uracil and D-ribose 5-phosphate in KEGG and in BioMeta**. Reaction entry R04457 from KEGG is shown with the problems indicated, including the product being shown on the left-hand side. The carbon and phosphorus imbalance causes the reaction to be unbalanced in oxygen and hydrogen as well. In BioMeta three reaction steps have been added to correctly represent this chemistry. Note that the reaction from D-ribose 5-phosphate to D-ribulose 5-phosphate (the reactant of MR005453) was already present in the database.

Riboflavin is biosynthesized from 6,7-dimethyl-8-(1-D-ribityl)-lumazine, which in turn is biosynthesized from 5-amino-6-(5-phosphoribitylamino)uracil and D-ribose 5-phosphate. The latter process is present in the KEGG ligand database as a single reaction (entry R04457, see Figure 8). This representation suffers from a number of problems, the most important being the imbalance in carbon, phosphorus, oxygen, and hydrogen. Moreover, the lumazine product is shown on the left-hand side of the reaction arrow. Since the actual process comprises four separate reaction steps[8], it seemed prudent to replace reaction entry R04457 by these four steps. In fact one of these steps (MR005453 in Figure 8) is already a quite complicated reaction by itself[29]. KEGG and BioMeta already contained the conversion of D-ribose 5-phosphate into D-ribulose 5-phosphate (KEGG entry R01056/BioMeta entry MR000958) so only the three reactions in Figure 8 had to be added to BioMeta.

#### Example 3

**Figure 9:**
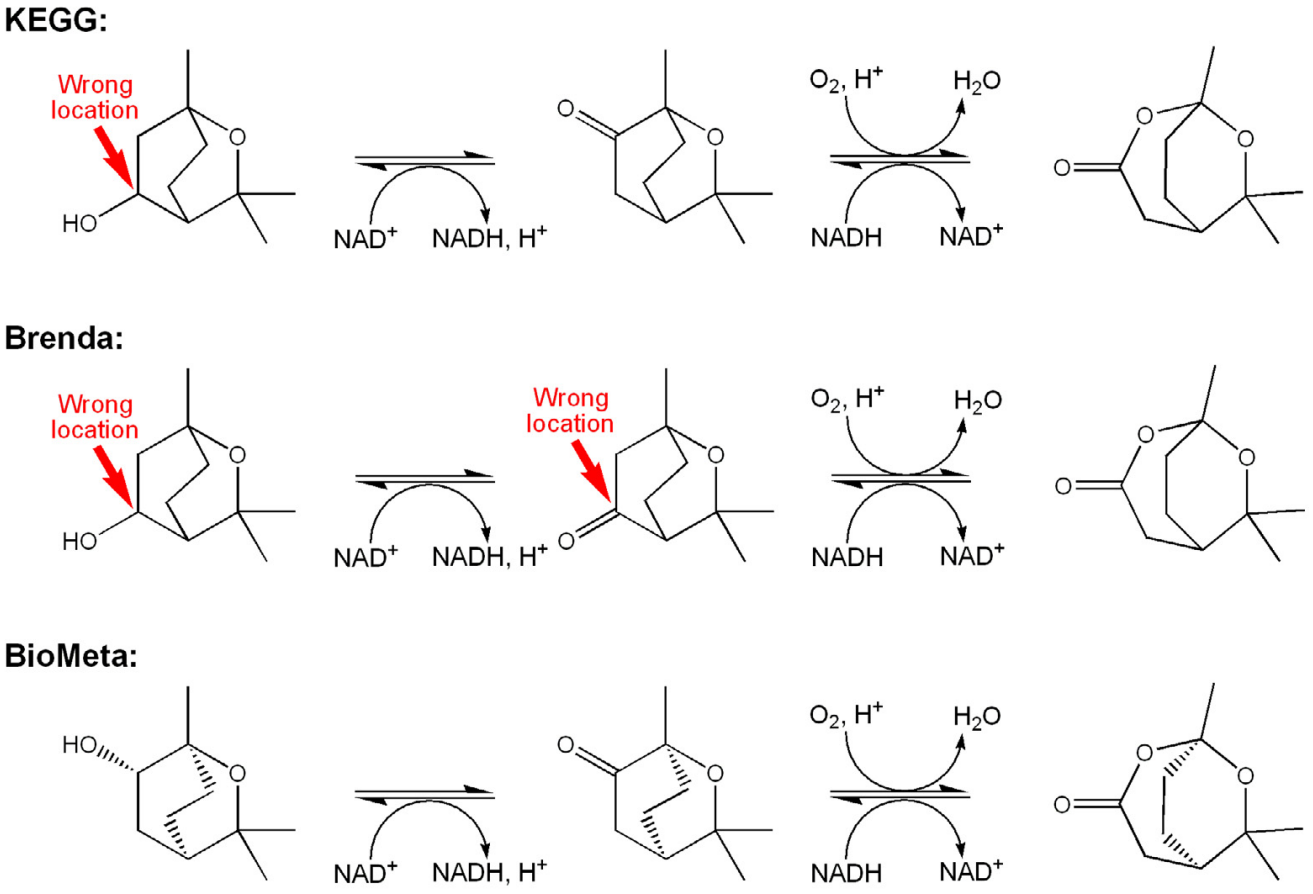
**The two reaction steps leading from (+)-2endo-hydroxy-1,8-cineole to (R, R)-1,6,6-trimethyl-2,7-dioxobicyclo-[3.2.2]nonan-3-one in the BioMeta, KEGG (entries R02994 and R02995), and Brenda databases**. Incorrectly positioned oxygen groups are indicated by red arrows. Note that the structures from both KEGG and Brenda lack stereochemistry.

The monoterpene 1,8-cineole is metabolized through (+)-2endo-hydroxy-1,8-cineole which in turn is degraded in two steps to (R, R)-1,6,6-trimethyl-2,7-dioxobicyclo-[3.2.2]nonan-3-one (Figure 9). The first of these steps looked rather odd in KEGG (entry R02994). A regular dehydrogenation by NAD^+ ^would be expected to produce a keto group at the same position as the original hydroxyl group. The same reaction in Brenda suggested that the ketone in KEGG was wrong, but now the next step, the oxygen insertion, looks very strange in Brenda. In KEGG this step (entry R02995) seems correct, a simple insertion of an oxygen into a C-C bond adjacent to a keto group (Baeyer-Villiger type oxidation). Further checking revealed[30] that in both databases the alcohol compounds were wrong and in Brenda the ketone as well. The compounds were corrected in BioMeta (Figure 9) with the correct stereochemistry[30].

### Database implementation details

The BioMeta database was implemented in PostgreSQL[31], an open-source relational database management system. Its contents are also stored in text (ASCII) files, and Python[32] scripts have been written to import these files into the database and to export the database contents into the text files. When the database is being filled, the output from the chemical validation software is included in the database import. The validation software has been written in Fortran. Python scripts have also been used for the web interface.

## Utility and Discussion

### Web interface

**Figure 10:**
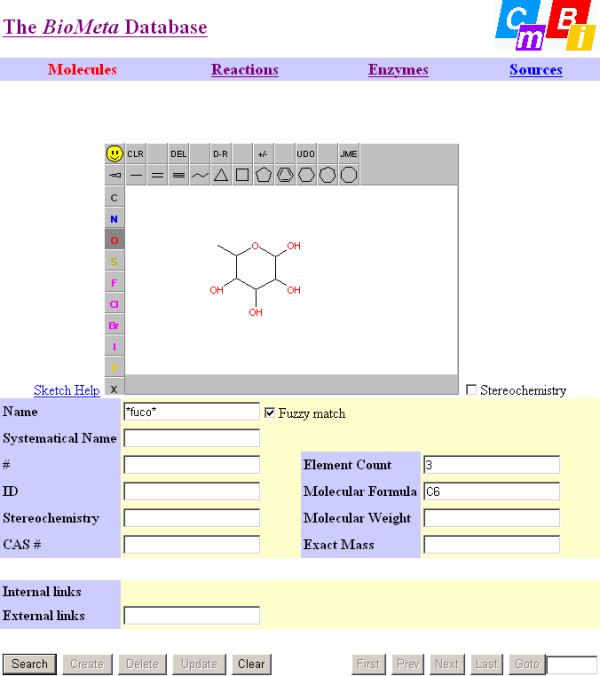
**A snapshot of the compound query menu of the BioMeta web interface**. The top row buttons lead to the four main search domains. The JME Molecular Editor is used to draw structure queries, optionally ignoring stereochemistry. The Name box allows searches for synonyms, optionally ignoring case and punctuation ("fuzzy" matching). ID and CAS # allow searches for BioMeta ID and CAS registry number, resp. Element Count and Molecular Formula allow extensive formula queries. The External links field allows searches for KEGG accession number. Note that all text fields allow queries using wildcards, all numerical fields (Element Count, Molecular Weight, and Exact Mass) allow comparison and range queries, and all query options can be combined.

**Figure 11:**
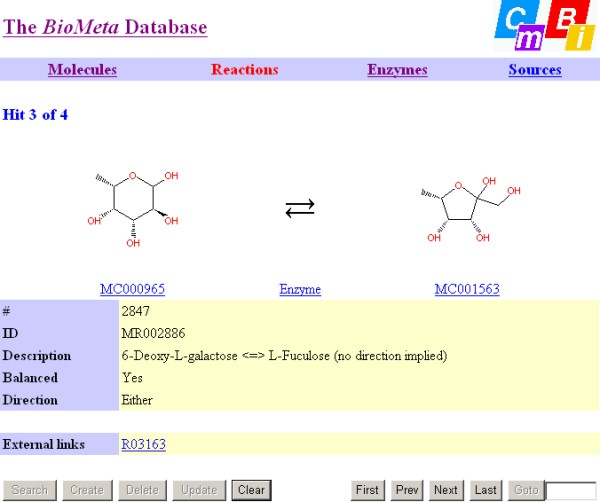
**The BioMeta web interface for reactions**. 'Description' contains the KEGG Definition string, 'ID' the BioMeta ID, 'Balance' the reaction balance (yes, no, or unknown) and 'Direction' the direction of the reaction (right, left, reversible, or unknown) which is also expressed by the reaction arrow. The substrates and products of the reaction can be directly accessed through hyperlinks, as can the enzyme(s) catalyzing the reaction. The hyperlink following 'external links' gives access to the corresponding KEGG reaction.

The database can be accessed through a web interface (Figures 10 and 11). Structures can be searched as exact structure (with or without stereochemistry taken into account), by name (with or without non-alphanumeric characters taken into account, called "fuzzy match" in the interface), by KEGG accession code, CAS registry number, molecular formula, molecular weight, or exact mass (calculated from the most abundant isotope for each element). A Java applet called JME (Java Molecular Editor)[33] is used to draw the structure queries (and to display structures from the database). All string fields allow substring searching using wildcards (asterisks), all numeric fields allow comparison and range searching (*e.g.*, molecular weight 123.2–123.9), and all search options can be combined in a logical "and" fashion. Name searches are conducted in the synonym tables. When a compound is displayed, a hyperlink is available to search for all reactions in which it is involved. Similarly, when a reaction is displayed hyperlinks are available to 1) search for all enzymes which catalyze it; and 2) access each molecule involved in the reaction, and when an enzymes is displayed a hyperlink is available to search for all reactions that it catalyzes. The interface allows to follow biochemical pathways quite quickly and efficiently, also because different browser windows are used for compounds, reactions, and enzymes.

In addition to the various data fields calculated from the structure, The web interface displays the various data fields calculated from the structures and the reaction, including the validation results. For compounds, the stereochemical information (field "Stereochemistry") is displayed with respect to completeness: "None" if the compound cannot exhibit stereoisomerism, "None (i.e., undefined)" if stereoisomerism is possible but stereochemistry is completely absent, "Meso" if the compound is achiral, "Relative" if the compound is chiral but a racemic mixture is indicated (this may or may not be intentional, drugs are often racemates), and finally "Absolute" if the compound is chiral and the enantiomer shown is the intended one. "Meso", "Relative", and "Absolute" may be followed by the remark "partially defined" if one or more stereocenters are undefined. For reactions, the field "Balanced" indicates whether the reaction is balanced or not. In case of an unbalanced reaction the word "No" is followed by a chemical formula representing the difference between the reactants and products). If one or more compounds have a polymeric structure or do not have a structure at all, the balance is displayed as "Unknown".

We expect that BioMeta will prove useful for querying and browsing biochemical pathways, to search connecting reaction paths between metabolites, and to view (calculated) three-dimensional models of the structures, to obtain reliable molecular data on metabolites, etc. Three-dimensional structures (calculated by Corina[34]) are already available for compounds with stereochemically completely defined structures. In the future, BioMeta may also provide the basis of several inference engines. For example, graph-theoretical approaches can be applied to determine pathways from series of individual enzymatic reactions[35].

## Conclusion

We demonstrate that the validation of metabolite structures and reactions is a feasible and worthwhile undertaking, and that the validation results can be used to trigger corrections and improvements to BioMeta, our metabolite database. BioMeta provides some tools for rational drug design, reaction searches, and visualization. The database will be useful for querying and browsing biochemical pathways, and to obtain reference information for identifying compounds, and for all other applications that require the underlying molecular data to be correct.

We have made our corrections available to KEGG and will keep doing so for the foreseeable future.

## Availability and requirements

The BioMeta database is freely available as a web service[21] provided the copyright notice of all original data is cited. The restrictions for use of the database are the same as those for the use of the KEGG Ligand database. Academic users may freely use the web site. Non-academic users may also use the web site as end users, but any form of distribution is not allowed.

The interface makes use of the JME (Java Molecular Editor)[33] to display structures and to draw structure queries, so the browser needs to be Java-enabled.

Project name: The BioMeta Database

Project home page: 

Browser requirements: Microsoft Internet Explorer works best, but other browsers (*e.g.*, Firefox) will function satisfactorily.

Programming language: Java (no version restrictions) for the JME applet and for Jmol[36] (to display 3D structures).

## Authors' contributions

MO wrote the manuscript, designed the BioMeta database and implemented it in PostgreSQL, built the web interface, developed the validation software, and carried out the improvements to the molecule and reaction data in BioMeta. GV provided the impetus for the research and contributed throughout by discussions, and by revising the manuscript. Both authors read and improved the final manuscript.
